# Quantitative immunohistochemical analysis of myeloid cell marker expression in human cortex captures microglia heterogeneity with anatomical context

**DOI:** 10.1038/s41598-020-68086-z

**Published:** 2020-07-16

**Authors:** Molly E. V. Swanson, Helen C. Murray, Brigid Ryan, Richard L. M. Faull, Mike Dragunow, Maurice A. Curtis

**Affiliations:** 1 Department of Anatomy and Medical Imaging, Faculty of Medical and Health Science, University of Auckland, Private Bag 92019, Auckland, New Zealand; 2 Centre for Brain Research, Faculty of Medical and Health Science, University of Auckland, Private Bag 92019, Auckland, New Zealand; 3 Department of Pharmacology and Clinical Pharmacology, Faculty of Medical and Health Science, University of Auckland, Private Bag 92019, Auckland, New Zealand

**Keywords:** Cellular neuroscience, Glial biology

## Abstract

Current immunohistochemical methods of studying microglia in the post-mortem human brain do not capture the heterogeneity of microglial function in response to damage and disease. We therefore investigated the expression of eight myeloid cell proteins associated with changes in function alongside Iba1. To study the myeloid cells we used immunohistochemistry on *post-mortem* human middle temporal gyrus sections from neurologically normal individuals. First we investigated co-labelling between the classical ‘activation’ marker, HLA-DR and each of the other markers of interest. Significant co-labelling between HLA-DR with CD206, CD32, CD163, or L-Ferritin was observed, although complete overlap of expression of HLA-DR with aforementioned markers was not observed. A qualitative assessment also demonstrated that perivascular macrophages expressed higher levels of the markers of interest we investigated than microglia, suggesting perivascular macrophages show a more phagocytic and antigen presentation state in the human brain. To determine whether the markers of interest were expressed in different functional states, the immunoreactivity for each marker was qualitatively assessed on microglial morphologies. Degenerating marker, L-Ferritin, was specific for dystrophic microglia. We demonstrate that microglial heterogeneity can be investigated in immunohistochemically stain post-mortem human tissue by integrating the single-cell abundance of proteins and cell morphology to infer function.

## Introduction

Microglia are the innate immune cells of the brain capable of responding to damage and disease. Because of the high abundance of stimuli in vivo, the variety of microglial reactions in the normal and diseased human brain make this population highly heterogeneous. Historically, these microglial reactions during damage and/or disease have been considered ‘activation’. However, this on/off terminology is being phased out as it does not reflect microglial heterogeneity^1^. We sought to investigate how the heterogeneity of microglial and perivascular macrophage reactions can be investigated in post-mortem human tissue, integrating the single-cell abundance of key proteins of interest and cell morphology to infer function.

We sought to investigate seven myeloid cell proteins in this study: P2RY12, TMEM119, CD74, CD206, CD32, CD163, and L-Ferritin. These proteins were chosen because they have previously been highlighted by single cell RNA sequencing studies, investigated in post-mortem human brain studies, we could demonstrate antibody specificity in human brain, or show a unique expression by microglia or perivascular macrophages (Supplementary Table 1)^2–26^. We investigated the expression of these myeloid cell proteins in the human brain by co-labelling them with the classical immunohistochemical ‘activation’ marker, human leukocyte antigen-DR isotype (HLA-DR), investigating their expression on microglia and perivascular macrophages, and identifying their expression on microglia with different morphologies.

**Table 1:** Semi-quantitative summary of marker abundance on myeloid cell populations.

Marker	Microglia	Perivascular Macrophage
P2RY12	+++	−
TMEM119	+++	−
HLA-DR	++	+++
CD74	+++	+++
CD206	−	+++
CD32	++	+++
CD163	+	++
L-Ferritin	++	−

Key: (−) not observed, ( +) low abundance, (+ +) moderate abundance, or (+ + +) high abundance in the myeloid cell population.

One of the most common immunohistochemical markers used to identify and quantify so-called microglial activation in the normal and diseased post-mortem human brain is HLA-DR^27–29^. HLA-DR is an antigen presentation molecule involved in the activation of the adaptive immune system. This function, along with high HLA-DR immunoreactivity being identified surrounding amyloid beta plaques in human Alzheimer’s disease brains, resulted in HLA-DR being classed as a marker of pan microglial ‘activation’ in immunohistochemistry studies^30,31^. This presents challenges when considering the heterogeneity of microglial reactions to damage and disease. Furthermore, HLA-DR is constitutively expressed by some resting microglia in the human brain and there is no evidence that HLA-DR is up-regulated through all forms of microglial reactions^32,33^. HLA-DR is only one of thousands of proteins that are differentially up-regulated during a microglial reaction to damage and/or disease and reflects only one function. We hypothesise that using multiple markers which reflect different myeloid cell functions will better capture the heterogeneity of microglial functions in studies of post-mortem human tissue. We quantified the co-labelling between each marker and HLA-DR to determine whether we could identify different microglial populations in the post-mortem human brain that likely had different functions in the brain. Determining the expression of this subset of proteins relative to the classical immunohistochemical activation marker allowed us to determine whether they are expressed by unique populations of myeloid cells that do not express HLA-DR.

To determine whether the markers of interest (MOIs) are upregulated on microglia that have diverse morphologies, we assessed MOI expression on the common microglial morphologies as a proxy for particular microglial functions. Morphological changes occur during microglial responses to damage and/or disease, and are hypothesised to increase functions to maintain brain homeostasis^33^. Five distinct microglial morphologies are readily identifiable: ramified, hypertrophic, dystrophic, rod, and amoeboid^25,33–42^. In previous studies of post-mortem human brain tissue, microglial morphologies have been used to infer activation state and function ^25,26,38,40^. However, it is important to note that microglial functions are not specific to a given morphology, but rather that morphologies can imply an increase in given functions. We investigated protein expression on different microglial morphologies to determine their expression throughout different microglial states indicative of different functions.

One consideration of this study was that microglia are not the only myeloid cells in the human brain that express the proteins being investigated. Perivascular macrophages (PVMs) are one border-associated macrophage population that reside in the perivascular space of the blood brain barrier^16,20,43–46^. While microglia and PVMs are both resident myeloid populations in the human CNS, they occupy different central nervous system compartments and are therefore different functional populations. Because the hypothesis of this study was that the proteins investigated can be used as markers of microglial activation and function in the brain, the expression pattern of these proteins on microglia and PVMs was examined.

By determining the expression pattern of proteins indicative of function on microglia and PVMs in post-mortem human tissue we have demonstrated the ability to identify myeloid cell heterogeneity in the human cerebral cortex with anatomical context. Our results provide a baseline for the co-labelling of these markers with HLA-DR in neurologically normal aged brains which establishes important context for subsequent studies of neurological disease.

## Results

### Markers of interest are differentially expressed in grey versus white matter across the Iba1-positive population

**Figure 1:**
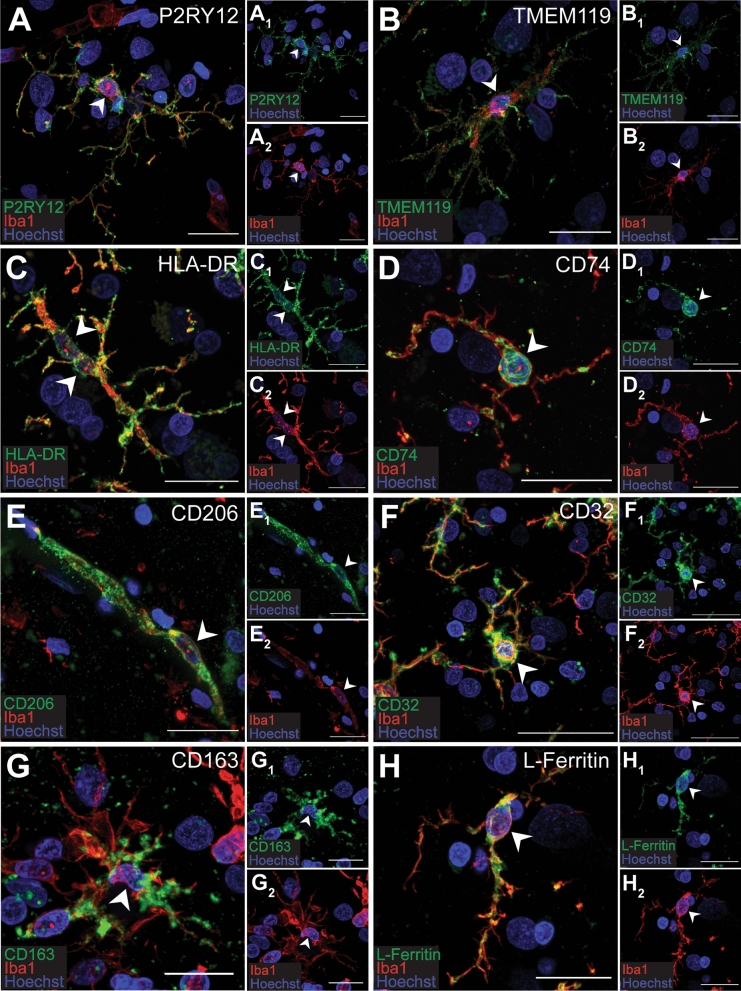
Marker of interest expression by Iba1-positive cells. MOIs, P2RY12 (**A**), TMEM119 (**B**), HLA-DR (**C**), CD74 (**D**), CD206 (**E**), CD32 (**F**), CD163 (**G**), and L-Ferritin (**H**), were fluorescently co-labelled with pan myeloid cell maker, Iba1, and Hoechst nuclear counterstain in 50-µm thick normal human middle temporal gyrus sections. Images are maximum projections of confocal *z*-stacks; scale bars = 20 µm. Arrows indicate Iba1-positive cell bodies with marker of interest immunoreactivity.

The immunoreactivity of the MOIs indicative of function were first assessed on Iba1-positive myeloid cells in the middle temporal gyrus grey matter (GM) and white matter (WM) from neurologically normal subjects. Immunoreactivity for P2RY12, TMEM119, HLA-DR, CD74, CD206, CD32, CD163, and L-Ferritin was observed on Iba1-positive cells (Fig. 1).

To investigate the expression of the MOIs within the myeloid cell population we used manual cell counting to determine the percentage of total Iba1-positive cells that were immunoreactive for each MOI. We carried out this quantification in both the GM and WM as previous research has not investigated the potential differences in the MOI expression between these regions. It is important to note that we deemed it appropriate to compare the abundance of MOIs normalised to the total number of Iba1-positive cells for this study because we did not identify any difference in Iba1-positive cell density between GM and WM (mean WM-GM difference = 17.16 ± 20.88 cells/mm^2^, p = 0.1001).

**Figure 2:**
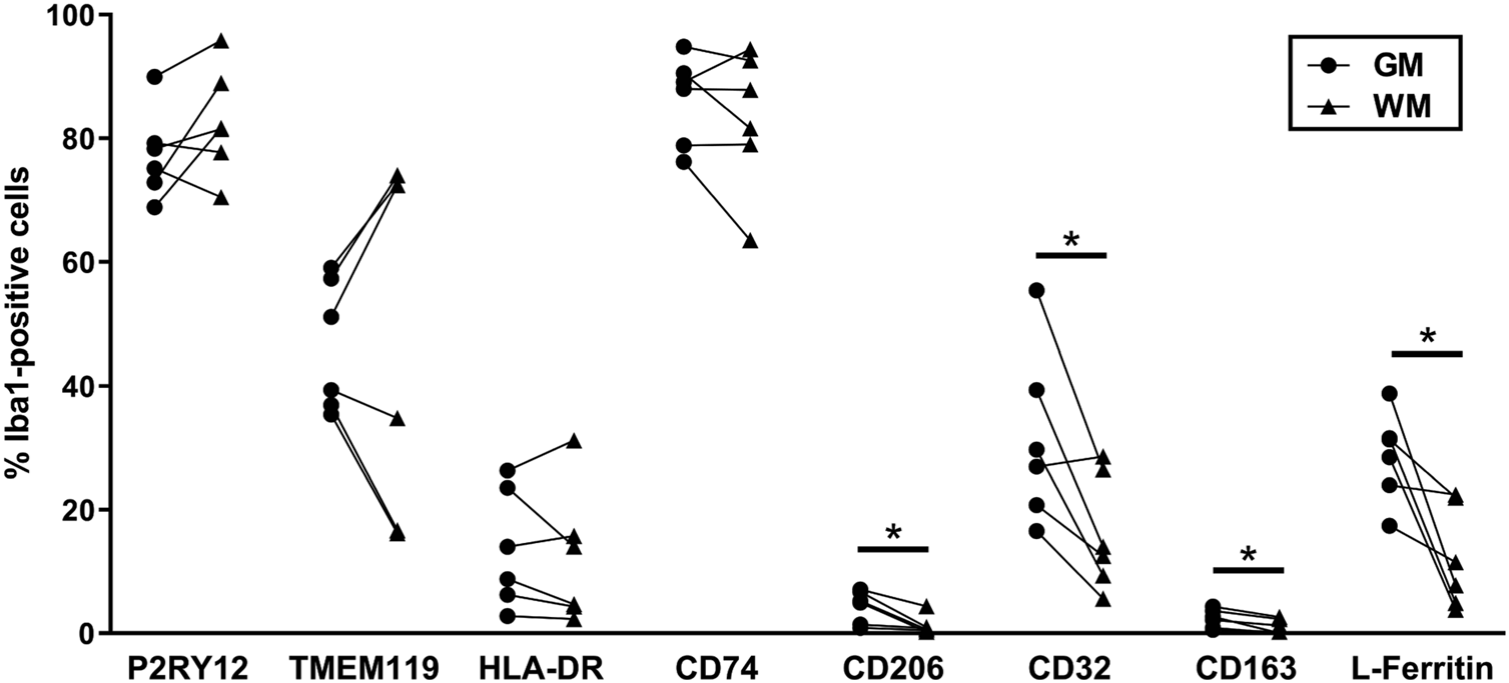
Quantification of the abundance of markers of interest on Iba1-positive cells. Iba1-positive cells were manually counted in the human middle temporal gyrus and the proportion immunoreactive for MOIs P2RY12, TMEM119, HLA-DR, CD74, CD206, CD32, CD163, or L-Ferritin was determined. The number of MOI-positive cells were normalized to the total number of Iba1-positive cells, and the percentages of MOI-positive cells in GM and WM were compared using paired t-tests. Data presented with each case represented by a single point and the percentages measured in GM and WM are joined per case (n = 6). Significant difference between GM and WM: *p < 0.05.

P2RY12 and CD74 were the most abundant markers in the Iba1-positive population and were equally distributed between GM and WM (Fig. 2). The two least abundant markers were CD206 and CD163, which were more highly expressed by Iba1-positive cells in GM than WM (Fig. 2). CD32 and L-Ferritin, were also more highly expressed by Iba1-positive cells in the GM than WM (Fig. 2). TMEM119 and HLA-DR, were expressed equally by Iba1-positive cells in the GM and WM (Fig. 2). The abundance of the MOIs in the Iba1-positive population did not correlate with age or post-mortem delay (Supplementary Fig. 2).

### Markers of interest are expressed by HLA-DR-positive Iba1-positive cells, but the cell-by-cell intensity of each marker does not strongly correlate with HLA-DR intensity

Quantification of the MOIs on Iba1-positive cells demonstrated that the percentage of Iba1-positive cells that expressed these proteins differed depending on the MOI. We hypothesised that this was because at least some of the MOIs were up-regulated by microglia with different predominant functions during reactions. We hypothesised that these MOIs could be expressed by functional populations not expressing HLA-DR. To test this hypothesis, we carried out a co-labelling analysis with HLA-DR. Only the GM was used for this aspect of the study and all Iba1-positive cells within the image were manually counted.

**Figure 3:**
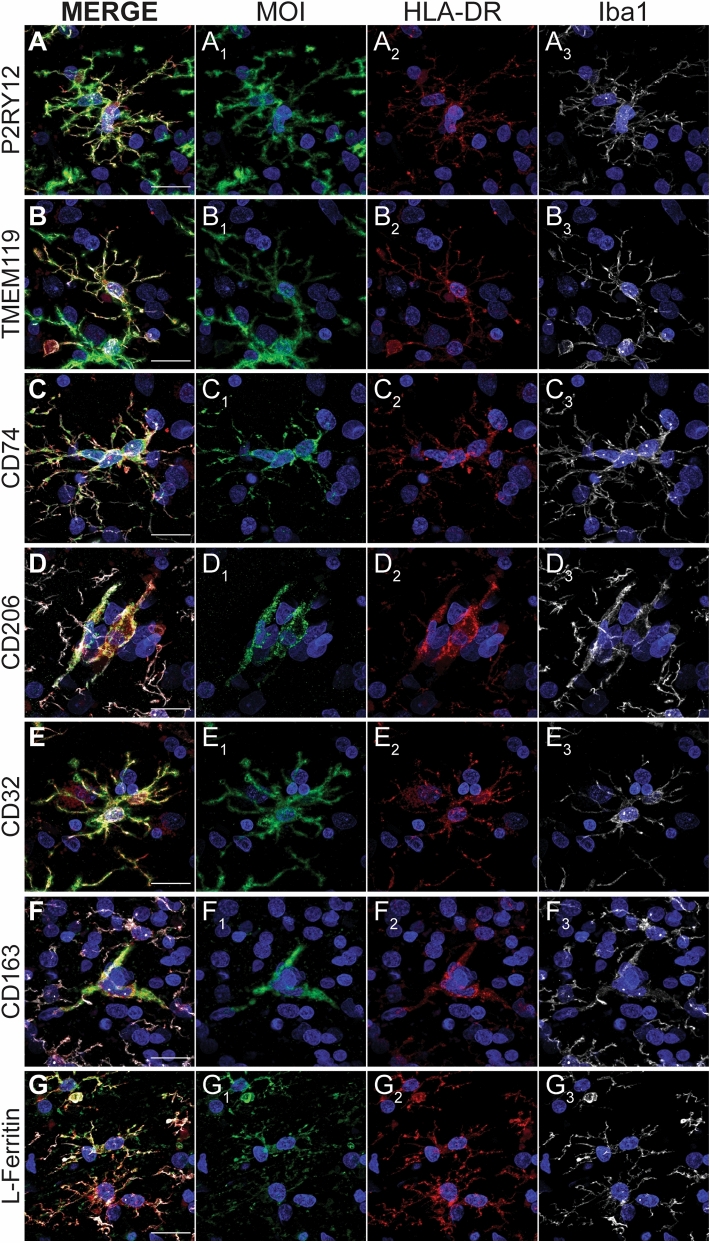
Immunofluorescent co-labelling of markers of interest with HLA-DR on Iba1-positive cells. MOIs, P2RY12 (**A**), TMEM119 (**B**), CD74 (**C**), CD206 (**D**), CD32 (**E**), CD163 (**F**), and L-Ferritin (**G**), were fluorescently co-labelled with HLA-DR, pan myeloid cell maker, Iba1, and Hoechst nuclear counterstain in 50-µm thick normal human middle temporal gyrus sections. Images are maximum projections of confocal *z*-stacks; scale bars = 20 µm.

**Figure 4:**
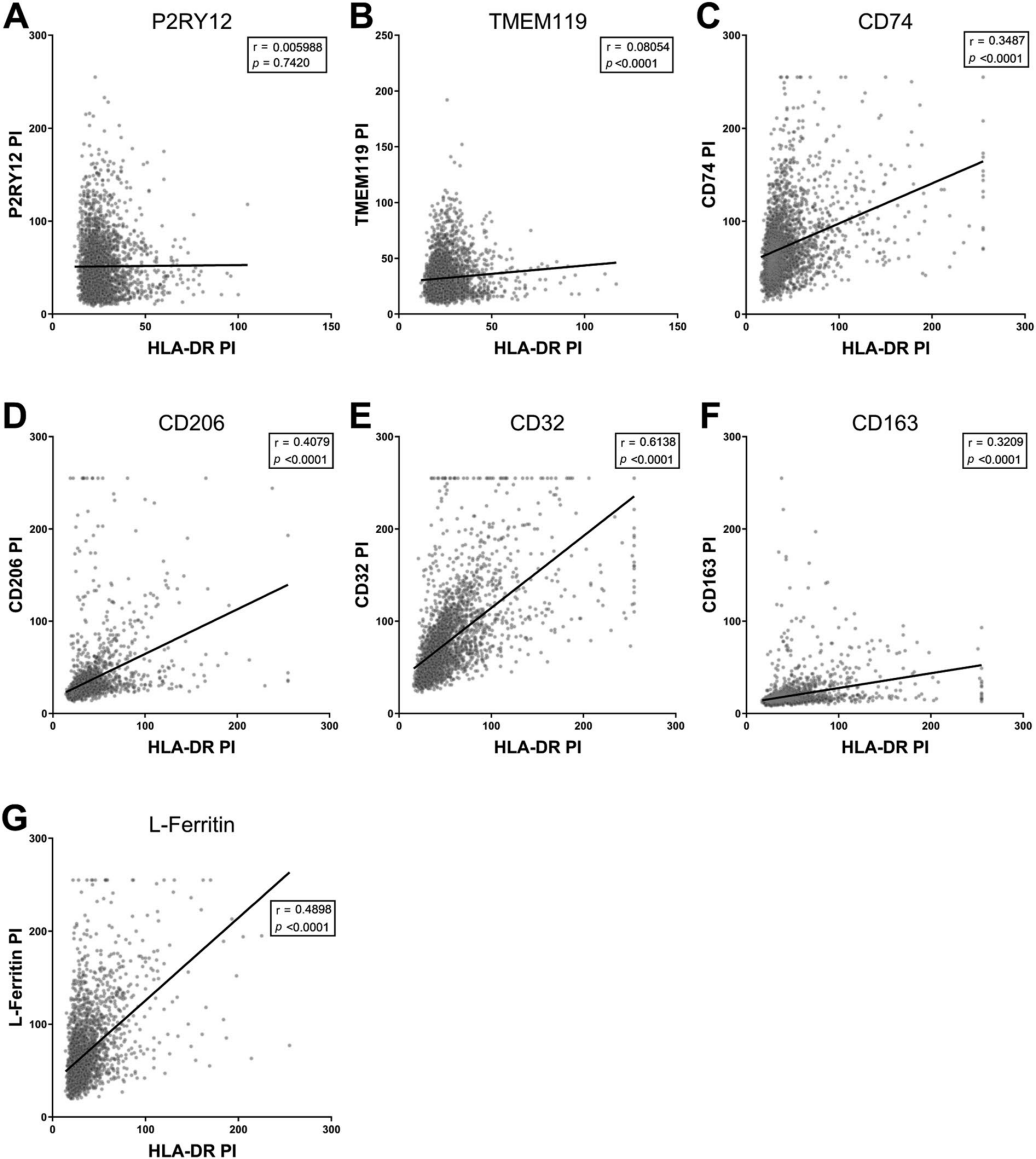
Correlation analysis of marker of interest and HLA-DR point intensities on Iba1-positive cells. One MOI was co-labelled with HLA-DR and pan myeloid cell marker, Iba1, in 10-µm thick normal human middle temporal gyrus sections. Iba1-positive cells in the grey matter were manually counted and the point intensities of the MOI and HLA-DR were measured. All Iba1-positive cell HLA-DR and MOI point intensities were pooled from all cases and Pearson’s correlations were used to determine whether the HLA-DR point intensity correlated with the point intensities of MOI P2RY12 (**A**), TMEM119 (**B**), CD74 (**C**), CD206 (**D**), CD32 (**E**), CD163 (**F**), or L-Ferritin (**G**). Abbreviation: PI, point intensity.

All the MOIs we studied co-labelled with HLA-DR (Fig. 3). To determine whether there was a relationship between HLA-DR and MOI expression, we investigated the correlation between the point intensities of HLA-DR and each MOI on all Iba1-positive cells (Fig. 4). No linear correlations between HLA-DR point intensities and TMEM119 or P2RY12 point intensities were observed on Iba1-positive cells (Fig. 4A, B). Only moderate correlations between HLA-DR point intensities and the remaining MOI point intensities were observed (Fig. 4C–G). Of these correlations, we further analysed the cells with HLA-DR or MOI point intensities in the top ten percent of values to determine whether the Iba1-positive cells that are potentially the most reactive are up-regulating multiple proteins and respective functions. When the top ten percent of HLA-DR point intensities were analysed, we did not observe any stronger correlations with the MOI point intensities (Supplementary Fig. 3). Furthermore, when the top ten percent of MOI point intensities were analysed, no stronger correlations with HLA-DR point intensities were observed (Supplementary Fig. 3). Therefore, the Iba1-positive cells that highly express HLA-DR do not highly express other MOIs indicative of other microglial functions.

### Categorisation of HLA-DR and MOI populations shows functional markers are more abundant on HLA-DR^high^ than on HLA-DR^low^ Iba1-positive cells

**Figure 5:**
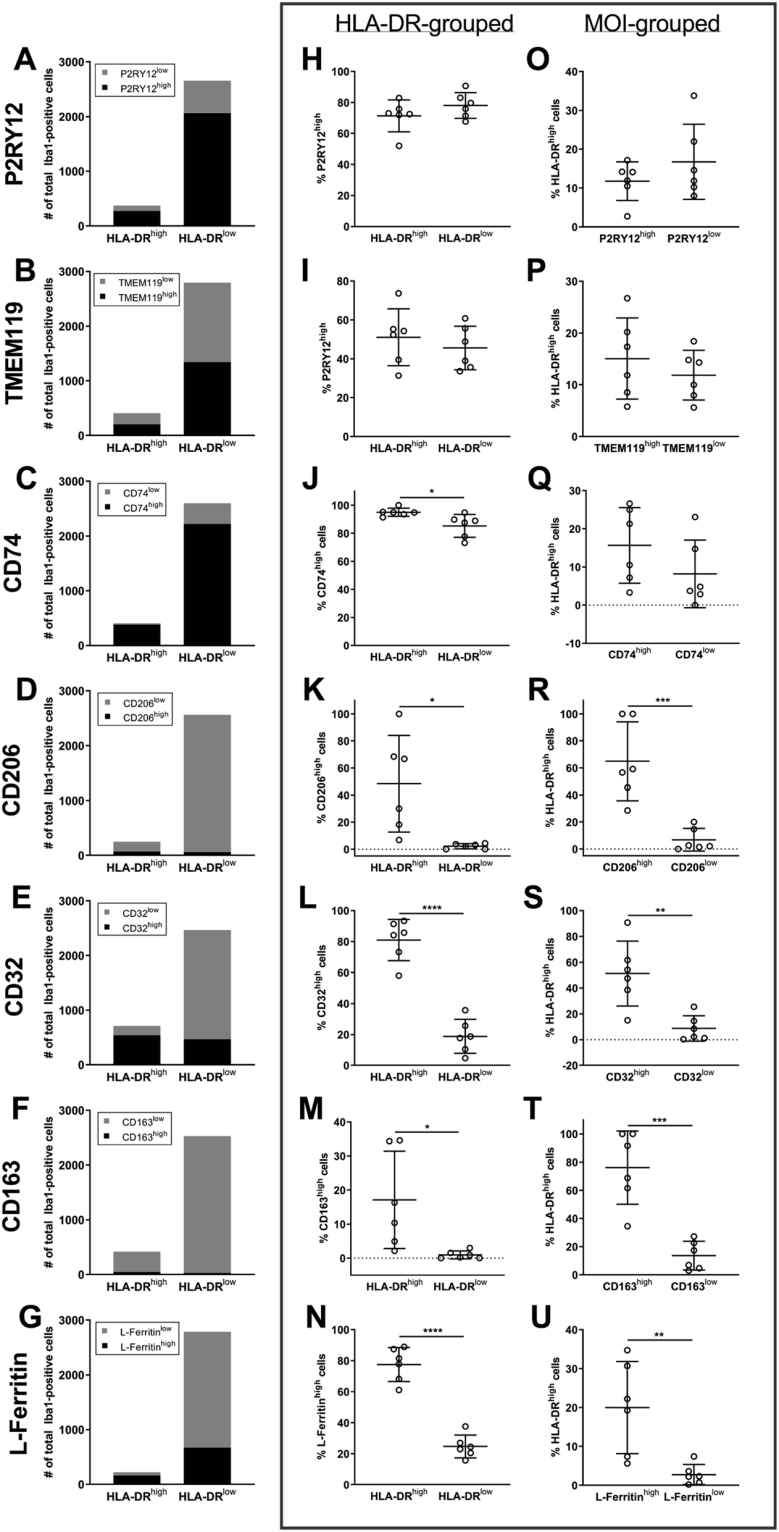
Distribution analyses of categorized HLA-DR and marker of interest high-low Iba1-positive populations. One MOI was co-labelled with HLA-DR and pan myeloid cell marker, Iba1, in 10-µm thick normal human middle temporal gyrus sections. The point intensities (PI) of HLA-DR and MOI P2RY12 (**A**, **H**, and **O**), TMEM119 (**B**, **I**, and **P**), CD74 (**C**, **J**, and **Q**), CD206 (**D**, **K**, and **R**), CD32 (**E**, **L**, and **S**), CD163 (**F**, **M**, and **T**), or L-Ferritin (**G**, **N**, and **U**) measured on Iba1-positive cells were used to categorize each Iba1-positive cell as either HLA-DR^high^ MOI^high^, HLA-DR^high^ MOI^low^, HLA-DR^low^ MOI^high^, or HLA-DR^low^ MOI^low^. Chi-square analyses were used to determine whether HLA-DR expression and MOI expression are independent of one another (**A**–**G**); data are presented as pooled Iba1-positive cells from the GM of all 6 normal cases. MOI^high^ cells were grouped based on their HLA-DR status, and the proportion of HLA-DR^high^ or HLA-DR^low^ cells determined to be MOI^high^ were compared with a student’s t-test (H–N); data are presented as mean ± SD (n = 6). HLA-DR^high^ cells were subsequently grouped based on their MOI status, and the proportion of MOI^high^ or MOI^low^ cells determined to be HLA-DR^high^ were compared with a student’s t-test (O–U); data are presented as mean ± SD (n = 6). Significance of differences between high-low populations: *p < 0.05, **p < 0.01, ***p < 0.001, ****p < 0.0001.

Some moderate linear correlations were observed in the overall correlations of HLA-DR with MOI point intensities on Iba1-positive cells. However, the highest ten percent of HLA-DR or MOI point intensity correlations demonstrated that high HLA-DR and high MOI expression did not always co-occur. This result may have simply been an artefact of HLA-DR and MOI point intensity variability across Iba1-positive cell bodies. To test this, we carried out a second analysis whereby we categorised Iba1-positive cells as HLA-DR^high/low^ and MOI^high/low^, resulting in four populations; HLA-DR^high^ MOI^high^, HLA-DR^high^ MOI^low^, HLA-DR^low^ MOI^high^, and HLA-DR^low^ MOI^low^. Chi-square analyses were used on the pooled Iba1-positive populations from all six normal cases to determine whether there was a relationship between the cellular abundance of HLA-DR and the MOIs (Fig. 5A–G). The percentage of MOI^high^ cells within the HLA-DR^high^ versus the HLA-DR^low^ populations was analysed to determine whether HLA-DR^high^ cells had a higher abundance of the MOI (Fig. 5H–N). Conversely, the percentage of HLA-DR^high^ cells within the MOI^high^ versus the MOI^low^ populations was analysed to determine whether the MOI^high^ population had a higher abundance of HLA-DR (Fig. 5O–U). This categorial analysis allowed us to determine whether the MOIs investigated in this study are highly expressed by Iba1-positive cells not expressing HLA-DR.

In accordance with the lack of linear correlations identified, a Chi-square analysis on the pooled Iba1-positive HLA-DR-P2RY12 and HLA-DR-TMEM119 populations showed no relationship between HLA-DR and the expression of P2RY12 or TMEM119 (Fig. 5A, B). Furthermore, when the populations were grouped by HLA-DR^high/low^, the percentage of the HLA-DR^high^ population identified as P2RY12^high^ or TMEM119^high^ was not statistically different from the percentage of the HLA-DR^low^ population that was P2RY12^high^ or TMEM119^high^ (Fig. 5H, I). The same result was observed when the populations were grouped by P2RY12^high/low^ or TMEM119^high/low^ and the percentage of these populations that were HLA-DR^high^ were compared (Fig. 5O, P). Therefore, neither microglial-specific marker is more abundant on HLA-DR^high^ Iba1-positive cells than on HLA-DR^low^ Iba1-positive cells.

Chi-square analysis on the pooled Iba1-positive HLA-DR-CD74 population demonstrated a relationship between HLA-DR and CD74 expression (Fig. 5C). When the HLA-DR-CD74 populations was grouped by HLA-DR^high/low^, we found that the percentage of the HLA-DR^high^ population that was CD74^high^ was significantly higher than the percentage of the HLA-DR^low^ population that was CD74^high^ (Fig. 5J). In contrast with this, when the HLA-DR-CD74 populations were grouped by CD74^high/low^, the percentage of the CD74^high^ population that was also HLA-DR^high^ was not significantly different to the percentage of the CD74^low^ population that was HLA-DR^high^ (Fig. 5Q). Therefore, while CD74 was more abundant in the HLA-DR^high^ population, HLA-DR was equally abundant in the CD74^high^ and CD74^low^ populations.

Because of the high expression of P2RY12, TMEM119, and CD74 across the entire Iba1-positive population and the unremarkable pattern of co-labelling with HLA-DR, these three MOIs are unlikely to reflect unique functional populations of reactive microglia in post-mortem human brain.

Chi-square analyses on the remaining pooled Iba1-positive HLA-DR-MOI populations demonstrated relationships between HLA-DR and the remaining MOIs (Fig. 5D–G). When the populations were grouped by HLA-DR^high/low^, we found that the percentage of the HLA-DR^high^ population that was MOI^high^ was significantly higher than the percentage of the HLA-DR^low^ population that was MOI^high^ (Fig. 5J–N). Therefore, these five MOIs were more abundant in the HLA-DR^high^ than the HLA-DR^low^ population. For the remaining MOIs, when the populations were grouped by MOI^high/low^, the percentage of the MOI^high^ population identified as HLA-DR^high^ was significantly higher than the percentage of the MOI^low^ that were HLA-DR^high^ (Fig. 5R–U). Therefore, we identified significant co-labelling between HLA-DR and CD206, CD32, CD163, or L-Ferritin in post-mortem human brain tissue. It is important to note that for all of these MOIs, we also identified HLA-DR^high^ MOI^low^ and HLA-DR^low^ MOI^high^ populations. So while HLA-DR^high^ Iba1-positive cells are more likely to also be MOI^high^ than HLA-DR^low^ Iba1-positive cells, some Iba1-positive cells uniquely express HLA-DR or a MOI highly.

### Markers of interest are differentially expressed by microglia and perivascular macrophages in the normal human middle temporal gyrus

**Figure 6:**
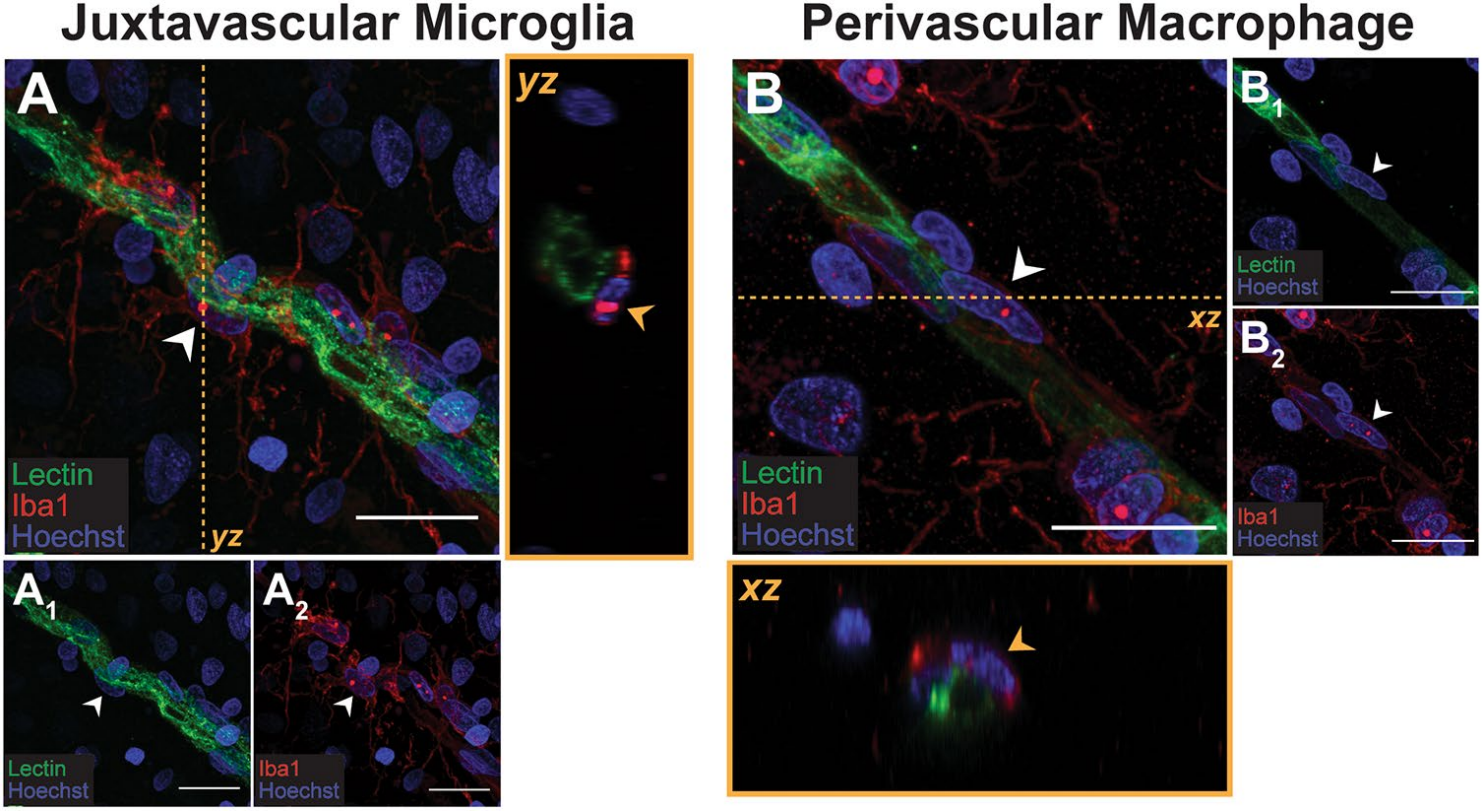
Anatomical location and morphologies of microglia and perivascular macrophages relative to lectin-positive blood vessels. Immunofluorescent double-labelling of pan myeloid cell marker, Iba1, with endothelial cell marker, lectin, with a Hoechst nuclear counterstain in 100-µm thick normal human middle temporal gyrus sections allowed for the visualization of juxtavascular microglia (**A**) and PVMs (**B**) and identification of cell characteristics. Juxtavascular microglia appeared as highly ramified Iba1-positive cell adjacent to the lectin-positive endothelial layer of blood vessels (**A**). The *yz* orthogonal view demonstrates that the Iba1-positive microglia lies outside of the blood vessel with no processes penetrating the blood vessel. PVMs appeared as elongated Iba1-positive cells devoid of processes with large elongated nuclei (**B**). The *xz* orthogonal view demonstrates the PVM lies adjacent to the blood vessel, not within it. Scale bars = 20 µm.

Our results so far have not distinguished between the different types of myeloid cells that express Iba1 in the human brain. Within the middle temporal gyrus grey matter, we would expect to encounter both Iba1-positive microglia and PVMs. The majority of microglia are not associated with blood vessels and they are therefore typically easily distinguishable from PVMs. However, a subset of microglia (juxtavascular microglia) are located alongside blood vessels and are therefore indistinguishable from PVMs by location alone. To overcome this issue, Iba1-positive cells were classified as microglia or PVMs based on their cellular morphology *and* location relative to lectin-positive blood vessels. Microglia were identified as Iba1-positive cells with a highly ramified morphology which could be juxtavascular i.e. associated with lectin-positive blood vessels (Fig. 6A), as well as scattered throughout the brain parenchyma. In contrast, PVMs were identified as Iba1-positive cells with an elongated cell body adjacent to lectin-positive blood vessels (Fig. 6B).

Using this method to identify microglia and PVMs, we investigated the marker of interest immunoreactivities on these CNS myeloid cells (Table 1). Based on the semi-quantitative assessment of the population wide expression, seven of eight MOIs investigated were differentially expressed by microglia versus PVMs. P2RY12, TMEM119, and L-Ferritin were only observed on microglia. Conversely, CD206 was only observed on PVMs. HLA-DR, M CD32, and CD163 were expressed by both microglia and PVMs but were more highly expressed by PVMs than microglia. CD74 was the only marker to be equally expressed by both myeloid populations.

### Marker of interest expression varies across microglial morphologies

The identification of high, but not total, co-occurrence of HLA-DR^high^ and MOI^high^ expression in the case of CD32, CD163, and L-Ferritin led to the hypothesis that each of these MOI are more up-regulated during different microglial reactions than HLA-DR. We hypothesise that high expression of HLA-DR or the MOIs investigated in this study are indicative of an increase in a distinct function during microglial reactions. One way of assessing microglial reactions in post-mortem human tissue is through the analysis of microglial morphologies. Therefore, to investigate this hypothesis, we qualitatively assessed the expression of HLA-DR and MOIs across different microglial morphologies.

**Figure 7:**
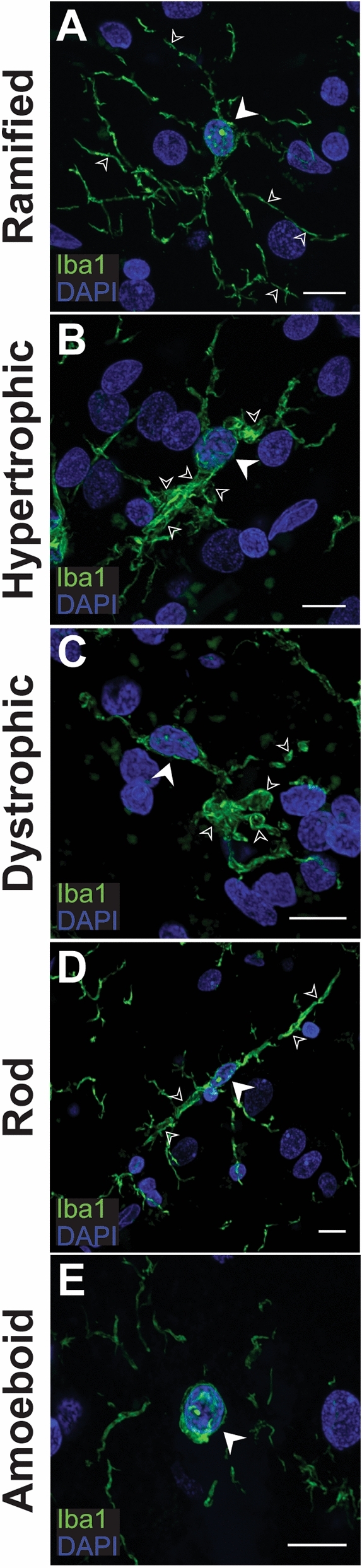
Indication of the five Iba1-positive microglial morphologies in the human brain. Microglia expressing pan marker, Iba1 (green), with a ramified (**A**), hypertrophic (**B**), rod (**C**), dystrophic (**D**) and amoeboid (**E**) morphology present in the human middle temporal gyrus. Main cell bodies with Hoechst-positive nuclei (blue) are indicated with block white arrows while morphology characteristics are indicated with small white arrow outlines. Images are maximum projections of confocal *z*-stacks; scale bars = 10 μm.

Five Iba1-positive cell morphologies were identified in the normal human brain (Fig. 7). Ramified had small triangular cell bodies with thin, highly branched processes (Fig. 7A). Hypertrophic reactive microglia had larger cell bodies with more intense Iba1 immunoreactivity, thickened processes, and were typically bipolar (Fig. 7B). Dystrophic microglia are the damaged or dying microglia and were identified by de-ramification of processes, membrane fragmentation, and had small, rounded or irregularly shaped nuclei (Fig. 7C). Rod microglia are hypothesised to be the supportive morphology, believed to form along neuronal axons in the grey matter to support signalling^38^. These were identifiable as bipolar microglia with thin, branching processes that lay parallel to neuronal axons projecting through cortical layers (Fig. 7D). Amoeboid microglia can functionally traverse through tissue and readily phagocytose large debris. Morphologically, they have no processes or in some cases, have a small leading process. In this study, they were most readily identified as cells with Iba1 immunoreactivity in a layer around the nucleus (Fig. 7E).

Following the identification of Iba1-positive microglial morphologies, the expression of MOIs was investigated across microglial morphologies. It is important to note that amoeboid microglia were not included in this study because, (1) amoeboid microglia and macrophages in the brain parenchyma (whether they are due to infiltration before death or a post-mortem artefact) are difficult to distinguish; and (2) amoeboid microglia are not abundant in the neurologically normal aged human brain and it was unlikely that a representative amoeboid microglia sample could be obtained from the sections stained. Furthermore, CD206 was not included in this analysis due to its immunoreactivity being confined to PVMs.

P2RY12, TMEM119, HLA-DR, and CD74 were expressed across all four morphologies investigated. CD32 was expressed on pure ramified, hypertrophic, and dystrophic morphologies. It was expressed by microglia that were bipolar and displayed correct cortical orientation characteristic of rod microglia, but these CD32 immunoreactive cells also showed signs of hypertrophy. Similarly, CD163, was only expressed on ramified, hypertrophic, and dystrophic microglia. Finally, L-Ferritin, was observed on purely dystrophic microglia. Any ramified or hypertrophic microglia that expressed L-Ferritin appeared to have a mixed morphology whereby they also showed signs of dystrophy. Furthermore, no rod microglia showed L-Ferritin immunoreactivity. These results are summarised in Table 2.

**Table 2:** Immunoreactivity of markers of interest on microglial morphologies.

Marker of Interest	Ramified	Hypertrophic	Dystrophic	Rod
P2RY12	✓	✓	✓	✓
TMEM119	✓	✓	✓	✓
HLA-DR	✓	✓	✓	✓
CD74	✓	✓	✓	✓
CD32	✓	✓	✓	✓*
CD163	✓	✓	✓	×
L-Ferritin	✓*	✓*	✓	×

Key: (×) not observed, (✓) observed, (✓*) observed on mixed morphologies.

## Discussion

This study has provided a comprehensive characterization of HLA-DR co-labelling with seven other myeloid cell proteins that contribute to microglial reactions in vivo. Using single cell immunohistochemical quantification methods, we have shown that these proteins identify additional reactive microglial populations to that of HLA-DR alone in post-mortem cortex from neurologically normal aged controls. Our results therefore reinforce the conclusion from single-cell RNA sequencing studies that microglial heterogeneity is extensive^4,7,48–50^. As such, we propose that anatomical studies of microglial changes in disease context using post-mortem human tissue should use this wider panel of panel of markers in conjunction with HLA-DR.

Current immunohistochemical methods of studying microglia in the post-mortem human brain do not capture the heterogeneity of microglial function changes in response to damage and disease. Even in recent studies, HLA-DR is the standard and often lone marker used to identify microglial reactivity^27–29^. Analysis of microglia in post-mortem tissue is also complicated by using methods that quantify the immunoreactivity across an area of tissue and therefore miss the subtlety of changes that occur at the single cell level. We therefore undertook an immunohistochemical analysis of seven markers whose expression by Iba1-positive cells can be used to infer a wider range of cell functions in the post-mortem human cortex. We also developed and validated a single cell quantification method to assess the correlation of marker intensity with HLA-DR intensity. Our results indicate that this combined approach of single cell immunohistochemical analysis using a range of markers provides a more comprehensive assessment of microglial heterogeneity in post-mortem tissue with anatomical context. This is evidenced by our assessment of grey and white matter where we show the diverse expression of these proteins across these two compartments, supporting the findings of previous single-cell RNA sequencing studies^50^. Studies of post-mortem tissue are inherently limited in that they reflect a single time point at the end of life and stage of disease. This makes it difficult to study dynamic processes such as microglial reactions. While single-cell RNA sequencing studies in human tissue have provided evidence of distinct microglial populations with unique expression signatures, these findings remain to be validated anatomically using immunohistochemistry. Our approach provides an effective means to capture this spectrum of microglial function changes with anatomical context and assess new microglial markers in future studies.

Our study investigated proteins that can be used to distinguish microglia and PVMs (TMEM119, P2RY12, CD206) or infer an increase in different myeloid cell functions such as antigen presentation (HLA-DR, CD74), phagocytosis (CD32, CD163) or degeneration (L-ferritin). These protein markers have previously been used in studies of human brain tissue and provided a reliable basis to investigate microglial reaction heterogeneity in relation to HLA-DR expression using our quantification method. We identified that all seven of these proteins or MOIs are expressed by HLA-DR-positive myeloid cells (determined by Iba1), but the cell-by-cell intensity of each marker does not strongly correlate with HLA-DR intensity. Categorisation into high vs low HLA-DR and MOI populations shows functional markers are more abundant on HLA-DR^high^ Iba1-positive cells. Therefore, our data suggest that these myeloid cell proteins are significantly co-expressed with HLA-DR on Iba1-positive cells and that the HLA-DR population encompasses a broad range of microglial reactions. However, we also identified both HLA-DR^high^ MOI^low^ and HLA-DR^low^ MOI^high^ Iba1-positive populations. This finding suggests that HLA-DR and each of the MOIs also label different functional populations of Iba1-positive cells in immunohistochemically stained post-mortem tissue. This finding is consistent with extensive microglial heterogeneity observed in single cell RNA sequencing studies and indicates there is considerable heterogeneity even within the HLA-DR population^4,7,48–50^.

To further corroborate the functional changes that are implied by increased expression of the myeloid cell proteins, we investigated the expression of the MOIs on microglial morphologies. Different microglial morphologies can be identified in immunohistochemically stained post-mortem human tissue and are hypothesised to reflect the predominant function of a given microglia^33,38,51^. An important limitation is that morphological changes are highly dynamic, and it is extremely difficult to define the morphology of cells that may be in transition between different states. Therefore, quantitative assessment of morphologies is highly subjective. Studies have attempted to overcome this subjectivity by developing unbiased computational analysis methods. Cell body size, number of processes, and process volume have been used to classify microglia into ramified, hypertrophic, dystrophic and amoeboid morphologies^52,53^. However, as no defined set of morphological criteria exist, quantification of these classifications is still highly subjective to user defined parameters^38^. While qualitative assessment of morphologies does not account for cells in transitional states, it is possible to identify the cells at the extreme ends of the transitional spectrum as distinct morphologies. We used this approach to identify whether our markers showed specificity to any distinct morphologies.

The majority of the MOIs investigated did not show specificity to a single morphology, indicating that their functional associations are not necessarily indicative of a specific morphological shift. This result indicates that heterogeneity in microglial reactions encompasses morphological diversity as well as protein expression. However, L-Ferritin immunoreactivity in the human brain was specific to dystrophic microglia. While the shift from ramified ‘resting’ morphology to a hypertrophic morphology is associated with microglial reactions, dystrophic morphology is thought to reflect senescence and degeneration^33,34,38,39,51,54,55^. The accumulation of dystrophic L-Ferritin^high^ microglia in the AD brain is hypothesised to be a result of disrupted iron homeostasis^25,26^, which is also disrupted in the normal ageing brain. Thus, the L-ferritin^high^ population we identified likely reflects age-related processes. L-Ferritin point intensities moderately correlated with HLA-DR point intensities on Iba1-positive cells and the Chi-square analysis demonstrated that L-Ferritin was more abundant on HLA-DR^high^ cells than HLA-DR^low^ cells. This suggests that the HLA-DR microglial population includes degenerating microglia and together our results support those of previous studies indicating that L-ferritin and dystrophic morphology can reliably distinguish this population of senescent or degenerating microglia in human tissue^25,26^.

As our study investigated the expression of myeloid cell proteins on Iba1-positive cells, our analysis included both PVMs and microglia. To determine the expression of each MOI across these two myeloid cell populations, and therefore the potential contribution of the PVMs to our results, we carried out a qualitative assessment to determine MOI expression across microglia and PVMs. We found that HLA-DR, CD206, CD32, and CD163 were more abundant on PVMs than microglia. These protein expression patterns have been observed previously, where CD206 and CD163 have been described as positive discriminators of PVMs^16,20,21,56,57^. We identified CD163 immunoreactivity on ramified Iba1-positive microglia which, in parallel with previous studies, demonstrates that microglia are also capable of up-regulating CD163, just to a lesser extent than PVMs^20–24^. The three MOIs that were more highly expressed by PVMs than microglia (CD206, CD32, CD163) have roles in antigen presentation and phagocytosis^15,18,22,30,58^. The expression patterns observed in this study therefore suggest that in the normal human brain PVMs show greater antigen presentation and phagocytic capabilities than microglia.

Protein expression by microglia and PVMs is driven by microenvironmental stimuli. It is therefore hypothesised that the different protein expression profiles of PVMs and microglia are a result of them populating different CNS compartments^14^. The borders between the brain and the periphery restrict the influx of potentially harmful peripheral components into the brain parenchyma, thus helping to maintain homeostasis^16,20,43–46^. In normal conditions, parenchymal microglia would have reduced environmental stimuli driving MOI protein expression. In contrast, PVMs function on the border of the brain and peripheral blood stream, surveying the influx of peripheral components into the CNS from the blood^59,60^. PVMs are therefore subjected to a range of environmental stimuli and their protein expression is directly influenced by changes in the periphery. PVMs are key border phagocytes with antigen presentation capabilities that regulate immune responses, and all these functions involve the up-regulation of proteins like those investigated^14,15,20–24,60–62^. While the work presented here is not primarily focused on PVM activation, the differences in MOI expression between microglia and PVMs do highlight that these difference cell types contribute to the overall myeloid cell heterogeneity that we wish to study in post-mortem tissue in a disease context in future studies.

We have presented evidence that single cell immunohistochemical analysis of seven different myeloid cell proteins can be used to identify unique functional populations that are not distinguished by HLA-DR alone. We show that these proteins are significantly associated with HLA-DR, but also label Iba1-positive cells without HLA-DR expression. Furthermore, these markers are expressed by microglia that show a range of morphologies which emphasizes that both protein expression and morphology reflect overall microglial heterogeneity. We also identified that these proteins are differentially expressed by microglia and PVMs in the human brain. Our results provide a baseline for the co-labelling of these markers with HLA-DR in neurologically normal aged brains which establishes important context for subsequent studies of neurological disease.

## Methods

### Human tissue processing

Post-mortem human brain tissue was obtained from the Neurological Foundation Human Brain Bank at the University of Auckland Centre for Brain Research. The human tissue was donated to the Brain Bank with consent from the donors’ families and its use in this project was approved by the University of Auckland Human Participants Ethics Committee. Eleven normal cases were used for this study (Table 3; average age = 78.27 ± 12.01 years, range = 61–98 years; average post-mortem delay = 18.91 ± 6.268 h, range = 12–32 h; 5 female:6 male). All normal cases had no history of cognitive deficits and their cause of death was unrelated to any neurological condition. The normal cases had age-related pathological changes, relating to a maximum Braak score of II or maximum CERAD score of A3B0C0 or equivalent, as determined by an independent pathological analysis ^63–65^.

**Table 3:** Human cases used for this study.

Fixation	Case number	Hemisphere	Age	Sex	*Post-mortem* Delay (h)
Formalin-fixed frozen	H169	Right	81	Male	24
	H187	Left	98	Female	15
	H196	Left	85	Male	15
	H241	Right	76	Female	12
	H243	Right	77	Female	13
Paraffin-embedded	H229	Right	88	Female	17
	H230	Right	57	Female	32
	H240	Right	73	Male	27
	H242	Right	61	Male	20
	H244	Right	76	Male	16
	H246	Right	89	Male	17

Human brains were obtained at autopsy and the right, and on occasion the left, hemisphere was fixed by perfusion of formalin through the cerebral arteries and subsequently dissected into blocks, cryoprotected, and frozen as previously described ^66^. Coronal Sects. (50- or 100-µm thick) of fixed-frozen middle temporal gyrus were cut from the normal cases indicated in Table 3 using a freezing sliding microtome and stored at 4 ºC in PBS containing 0.1% sodium azide. These free-floating sections were used for the identification of MOI expression on Iba1-positive cells, qualitative co-labelling of each MOI with HLA-DR, the identification and qualitative assessment of MOI abundance on microglia and PVMs, and the assessment of MOI abundance on microglial morphologies.

During the dissection of the formalin-fixed hemisphere, 1-cm thick blocks were taken and processed for paraffin embedding as previously described^66^. For the quantitative co-labelling analysis of each MOI with HLA-DR, 10-μm thick sections were cut from paraffin-embedded middle temporal gyrus blocks from the normal cases indicated in Table 3 using a rotary microtome.

### Fluorescent immunohistochemical staining of Iba1 and markers of interest

For qualitative MOI assessments, free-floating fluorescent immunohistochemistry was carried out on the 50- and 100-µm thick middle temporal gyrus sections as previously described ^67^. In short, sections were placed into tris–EDTA pH 9.0 buffer and microwaved at 1,100 W for 30 s or until the solution boiled. The sections were incubated in the solution containing primary antibodies for 72 h at 4 °C on a rocking platform (Table 4) and then in a solution containing fluorophore-conjugated secondary antibody, specific to the primary antibody species, overnight at room temperature. Lastly, sections were incubated in Hoechst (1:20,000 in PBS, Molecular Probes, #33,342) mounted onto glass slides and coverslipped.

**Table 4:** Primary antibodies, concentrations, and visualisation methods for this study.

Primary antibody	Company, Catalogue number	Free-floating Immunohistochemistry		Paraffin Immunohistochemistry	
		Concentration	Visualisation	Concentration	Visualisation
goat anti-Iba1	Abcam, ab5076	1:2,000	Alexa Fluor 647 secondary	1:1,000	Alexa Fluor 647 secondary
rabbit anti-P2RY12	Sigma Aldrich, HPA014518	1:2,000	Alexa Fluor 488 secondary	1:500	TSA Alexa Fluor 488
rabbit anti-TMEM119	Abcam, ab185333	1:500	TSA Alexa Fluor 488	1:500	TSA Alexa Fluor 488
mouse anti-HLA-DR	DAKO, M0775	1:1,000	Alexa Fluor 488 or 594 secondary	1:2,000	Alexa Fluor 594 secondary
rabbit anti-CD74	Abcam, ab64772	1:500	Alexa Fluor 488 secondary	1:1,000	Alexa Fluor 488 secondary
rabbit anti-CD206	Abcam, ab64693	1:1,000	Alexa Fluor 488 secondary	1:2,000	Alexa Fluor 488 secondary
rabbit anti-CD32	Abcam, ab155972	1:500	TSA Alexa Fluor 488	1:2,000	Alexa Fluor 488 secondary
rabbit anti-CD163	Abcam, ab182422	1:1,000	TSA Alexa Fluor 488	1:1,000	Alexa Fluor 488 secondary
rabbit anti-L-Ferritin	Sigma Aldrich, F5012	1:2,000	Alexa Fluor 488 secondary	1:5,000	Alexa Fluor 488 secondary

Lectin from *Ulex europaeus-1* (*UEA-1*) was used for the visualisation of blood vessels as it is a specific marker of endothelial cells^68,69^. Two forms of lectin from *UEA-1* were used in conjunction with other primary antibodies to label blood vessels in the free-floating MTG sections. A biotinylated lectin from *UEA-1* (1:1,000, Sigma-Aldrich, L8262) was visualised using an AlexaFluor 647-conjugated Streptavidin (1:500, Sigma-Aldrich, S21374). Alternatively, a DyLight 594-conjugated lectin from *UEA-1* (1:100, Vector Laboratories, DL-1067) was used to directly visualise endothelia.

For the quantification of MOI co-labelling with HLA-DR, paraffin immunofluorescent staining was carried out on the 10-µm thick middle temporal gyrus sections as previously described^70^. In short, following dewaxing rehydration, antigen retrieval was carried out using tris–EDTA pH 9.0 buffer. Serum blocking was carried out and sections were subsequently incubated in the primary antibodies overnight at 4 ºC and secondary antibodies for 4 h at RT. Finally, sections were incubated in Hoechst (1:20,000 in PBS, Molecular Probes, #33,342) for 10 min to stain all nuclei and coverslipped.

### Tyramide signal amplification

A selection of primary antibodies required tyramide signal amplification (TSA) for visualisation as indicated in Table 2. TSA was carried out as previously described ^67^. The secondary antibody cocktail used contained donkey anti-rabbit biotinylated secondary antibody (1:5,000; Jackson Laboratories, 711–065-152) as well as the AlexaFluor conjugated secondary antibodies, specific to the non-amplified primary antibodies.

### Imaging

For the visualisation of MOIs on Iba1-positive myeloid cells and assessment of MOI abundance on different microglial morphologies, goat-Iba1 was co-labelled with one MOI antibody in 50-µm thick free-floating middle temporal gyrus sections. For identification of MOI co-labelling with HLA-DR, goat anti-Iba1 and mouse anti-HLA-DR were co-labelled with one MOI antibody in 50-µm thick free-floating middle temporal gyrus sections. All sections were imaged on the Olympus FV1000 confocal microscope using the 100 × oil immersion lens. Optical z-stacks were taken through the entirety of the cell body and processes. In the case of the microglial morphology assessments, morphologies were initially determined from the MOI staining, but also confirmed after imaging of both the MOI and Iba1. All images presented are focused maximum projections of confocal *z*-stacks of six to ten 1-μm thick optical slices.

To classify Iba1-positive cells as microglia or PVMs, goat anti-Iba1 was fluorescently co-labelled with lectin from *UEA-1* in the 100-µm thick middle temporal gyrus sections. Microglia and PVMs were first classified based on previously described cell morphologies and their location relative to lectin-positive blood vessels: parenchymal microglia were identified in the tissue parenchyma and had small cell bodies with ramified processes, while PVMs were identified alongside lectin-positive blood vessels and had elongated cell bodies. The parenchymal microglia included juxtavascular microglia, which are microglia associated with blood vessels. This allowed for a more direct comparison between microglia and PVMs with relation to blood vessel structures and immunoreactivity of MOIs. Both microglia and PVMs were imaged on the Olympus FV1000 confocal microscope using the 100 × oil immersion lens. Optical z-stacks at 1 μm intervals were taken though the entirety of the Iba1-positive cell body and its processes, as well as the entirety of the associated blood vessel. These optical z-stacks were presented as *z*-projections of the *xy* image to give a representation of the Iba1-positive cell and its adjacent vessel. Iba1 and UEA-1 were subsequently co-labelled with one MOI in 50-µm thick MTG sections to assess microglial and PVM expression of MOIs using the same imaging methods.

For the quantification of the abundance of MOIs co-labelled with HLA-DR, goat anti-Iba1 and mouse anti-HLA-DR were co-labelled with one MOI antibody in 10-µm thick paraffin-embedded MTG sections. Sections were imaged at 20 × magnification on a Zeiss Z2 Axioimager using MetaSystems VSlide acquisition software and MetaCyte stitching software. Acquired images were opened on VSViewer v2.1.112 and images from two areas of grey matter (GM) and two areas of white matter (WM) were extracted.

### Manual counting and measurement of marker of interest abundance

Manual counting was carried out in ImageJ v 1.5i. A region of interest (ROI) was drawn based on the Hoechst channel using the polygon selection tool. By selecting the counting ROI on the Hoechst channel, any bias towards areas of high or low co-labelling was removed. For the GM images, cortical layers I through VI were all included to exclude any layer bias. This ROI was approximately 1 mm^2^ for WM images and 1.5 mm y^2^ for GM images. Prior to manual counting, five point measurements of background were measure in the Iba1, MOI, and HLA-DR images. All Iba1-positive microglia were subsequently counted within this ROI using the multipoint region tool on an Iba1-Hoechst image. Each point was placed on the cell body of an Iba1-positive cell with a Hoechst-positive nucleus and was therefore considered a coordinate where a microglia was found. Point intensities of Iba1, HLA-DR, and the MOI were measured as grey values between 0 and 255 at each coordinate. To be considered Iba1-positive, the Iba1 point intensity needed to be 20 grey values above background.

#### Validation of point intensities as an accurate measure of Iba1, HLA-DR and MOI abundance in each cell.

To validate that the intensity of a single pixel within the Iba1-positive cell body was a sufficient measure of overall intensity, another 10 multipoint regions were placed randomly around the Iba1 cell body. The intensities of HLA-DR, CD74, or CD32 were subsequently measured at these 10 coordinates. The validation was carried out on approximately 30 Iba1-positive cells per case: a total of 148 cells were measured across the six cases. The mean point intensity measured across these 10 points were correlated with the original point intensity measured per Iba1-positive cell (Supplementary Fig. 1). The linear correlations were considered weak if r ≤ 0.3, moderate if 0.3 ≤ r ≤ 0.8 and strong if r ≥ 0.8, and statistical significance was set at p ≤ 0.05—the interpretation of the correlation values was used for all such correlations in this study.

For both cell membrane markers, the mean point intensity strongly correlated with the original measured PI (HLA-DR: r = 0.8601, p < 0.0001, Fig. 1A; CD32: r = 0.843, p < 0.0001, Fig. 1B). Alternatively, for the cytoplasmic marker, the measured point intensity only moderately correlated with the original measured PI (CD74: r = 0.7695, p < 0.0001, Fig. 1C) suggesting that single-pixel point intensity did not consistently reflect total cell intensity. For this reason, the single point intensity method was used in combination with a second method (categorisation), on the basis that a dual approach would strengthen the robustness of the conclusions. Therefore, multiple methods were employed to quantify and statistically analyse the abundance of HLA-DR and MOIs, and the extent of their co-labelling, in the Iba1-positive cell population.

#### Statistical analysis of MOI abundance in the Iba1-positive cell population.

If a cell was Iba1-positive, it was subsequently determined to be immunoreactive for HLA-DR or the MOI if the point intensities were 20 to 40 grey values above background. This grey value threshold was determined from the counting images, and it allowed for the detection of HLA-DR or MOI immunoreactivity within Iba1-positive cells and excluded autofluorescence present in the tissue. The mean percentages of Iba1-positive cells immunoreactive for HLA-DR or MOI measured in each case in GM and WM were compared using paired t-tests. Each data point represents a case. Statistical significance was set at p ≤ 0.05. These percentages were correlated with post-mortem delay and age using a Spearman’s correlation. Male–female differences could not be investigated because of the low number of cases in each group.

### Statistical analysis of HLA-DR and MOI co-labelling in the Iba1-positive cell population

Due to the variability in HLA-DR and MOI staining across Iba1-positive cell bodies, the statistical analysis of this co-labelling was carried out in two ways. Firstly, the point intensities of HLA-DR and MOI were linearly correlated. Secondly, The HLA-DR-MOI populations were categorised into HLA-DR^high/low^ and MOI^high/low^ and the distribution of Iba1-positive cells into each population was analysed using a Chi-square analysis. The rationale for categorisation was based on the identified limitation of using single pixel point intensities: if single pixel point intensity did not accurately reflect total cell intensity, binarization of the data would allow robust categorisation of a cell as expressing each marker either at a high or low level.

#### HLA-DR-MOI point intensity correlation analysis

To determine whether the HLA-DR and MOI expression was related, all Iba1-positive cells from all six normal cases were pooled and the HLA-DR and MOI point intensities were correlated using a Pearson’s correlation. To determine whether HLA-DR and MOI expression were related at higher levels of expression, the 10% of Iba1-positive cells with the highest HLA-DR point intensity or MOI point intensity were correlated with their respective MOI point intensity or HLA-DR point intensity with a Pearson’s correlation. While efforts were made to prevent overexposure of any pixels during imaging, some saturated pixels of 255 grey values were measured for HLA-DR or MOI point intensities. To ensure these point intensities did not skew any correlations carried out above, correlations were carried out with and without the saturated pixels. No differences in correlation strength or statistical significance were observed (data not shown).

#### Categorisation of HLA-DR-MOI populations and Chi-square analysis

To quantify the extent of HLA-DR and MOI co-labelling on microglia, Iba1-positive cells were categorised into HLA-DR^high/low^ and MOI^high/low^ groups and the distribution of cells in each HLA-DR-MOI population was statistically analysed. Using the original images, intensities for high versus low HLA-DR or MOI staining were compared to background staining. A threshold intensity above background was subsequently determined for HLA-DR and each MOI. If a point intensity for any given marker was higher than the threshold intensity above background, an Iba1-positive cell was considered to have a high expression for this marker. Alternatively, if the point intensity was lower than the threshold intensity above background, and Iba1-positive cell was considered to have a low expression. Therefore, each Iba1-positive cell was classified as HLA-DR^high/low^ and MOI^high/low^, resulting in four HLA-DR-MOI groups; HLA-DR^high^ MOI^high^, HLA-DR^high^ MOI^low^, HLA-DR^low^ MOI^high^, and HLA-DR^low^ MOI^low^.

HLA-DR and MOI co-labelling on Iba1 cells was first statistically assessed using a Chi-Square analysis. This test determined whether the distribution of Iba1 cells in the HLA-DR^high^ MOI^high^, HLA-DR^high^ MOI^low^, HLA-DR^low^ MOI^high^, and HLA-DR^low^ MOI^low^ groups was due to chance. Put simply, this test determined whether there was a relationship between HLA-DR expression and MOI expression. Statistical significance was set at p ≤ 0.05. Data presented are from the tests on pooled Iba1-positive cells from all cases. However, the distribution of HLA-DR-MOI populations in each case was also investigated and the same distributions and statistical significances were observed (data not shown).

To determine whether activated Iba1-positive cells were more likely to express MOIs, Iba1-positive cells from each normal case were grouped into HLA-DR^high^ or HLA-DR^low^ populations and the percentage of these populations found to be MOI^high^ was compared. To determine whether MOI^high^ Iba1-positive cells were more activated, Iba1-positive cells were grouped into MOI^high^ or MOI^low^ populations, and the percentage of these populations found to be HLA-DR^high^ was compared. Because all data were equally distributed with no difference in variances, data were compared using an unpaired student’s t-test and statistical significance was set at p ≤ 0.05.

### Ethics approval and consent to participate

Ethics approval was obtained through the University of Auckland Human Participants Ethics committee (protocol number 011654).

### Consent for publication

Not applicable.

## Supplementary information

Supplementary file1 (DOCX 5998 kb)

## Data Availability

Not applicable.

## Abbreviations

CD: Cluster of differentiation
CNS: Central nervous system
GM: Grey matter
HLA-DR: Human leukocyte antigen, DR isotype
Iba1: Ionised calcium binding adaptor protein 1
MOI: Marker of interest
PI: Point intensity
PMD: Post-mortem delay
PVM: Perivascular macrophage
TSA: Tyramide signal amplification
UEA-1: Ulex europaeus-1
WM: White matter
