# Supplementary material for: Quantitative immunohistochemical analysis of myeloid cell marker expression in human cortex captures microglia heterogeneity with anatomical context
Source: Sci Rep. 2020 Jul 16;10:11693. doi: 10.1038/s41598-020-68086-z (PMC7366669; doi:10.1038/s41598-020-68086-z)
Supplement: Supplementary file 1 — Supplementary file1 (DOCX 5998 kb) [file 41598_2020_68086_MOESM1_ESM.docx]

**SUPPLEMENTARY FIGURES FOR**

**Quantitative immunohistochemical analysis of myeloid cell marker expression in human cortex captures microglia heterogeneity with anatomical context**

Molly E. V. Swanson^1,2^, Helen C. Murray^1,2^, Brigid Ryan^1,2^, Richard L. M. Faull^1,2^, Mike Dragunow^2,3^, Maurice A. Curtis^1,2,4^

^1^ Department of Anatomy and Medical Imaging, Faculty of Medical and Health Science, University of Auckland, Private Bag 92019, Auckland, New Zealand

^2^ Centre for Brain Research, Faculty of Medical and Health Science, University of Auckland, Private Bag 92019, Auckland, New Zealand

^3^ Department of Pharmacology and Clinical Pharmacology, Faculty of Medical and Health Science, University of Auckland, Private Bag 92019, Auckland, New Zealand

| **Supplementary Table 1:** Markers of interest chosen to study in this study | | |
| --- | --- | --- |
| **Marker** | **Function** | **Reason to characterize** |
| P2RY12 | Detection of extracellular nucleotides^2^ | Microglial-specific marker^3^; reduced in disease-associated microglial population^4^; reduced expression by acutely active microglia^5,6^; enriched in some single cell RNA-sequencing populations^7^ |
| TMEM119 | Unknown | Microglial-specific marker^8,9^; reduced in disease-associated microglial population^4^ |
| CD74 | Supports HLA-DR formation and transportation to the cell surface^10,11^ | Enriched in an Alzheimer’s disease pathology-associated microglial population^7^; CD74-positive cells associated with tau tangles in Alzheimer’s disease^12^ |
| CD206 | Phagocytosis and endocytosis of mannosylated enzymes and proteins^13^ | Perivascular macrophage-specific marker^14–16^ |
| CD32 | Bind to Fc regions of antibodies to aid in phagocytosis^17^ | Increased number of CD32-positive cells associated with amyloid beta plaques in Alzheimer’s disease^18^ |
| CD163 | Clears extracellular haemoglobin-haptoglobin complexes, and binds and sequesters TNFα-like weaker inducer of apoptosis^19^ | High expression by perivascular macrophages in the normal human brain^20,21^; increased expression by activated hypertrophic microglia in the normal, Alzheimer’s and Parkinson’s disease brain^22–24^ |
| L-Ferritin | Sequesters free iron to reduce the formation of reactive oxygen species | Expressed by dystrophic microglia in the human brain^25^; immunoreactivity precedes formation of tau tangles in Alzheimer’s disease^26^ |

**
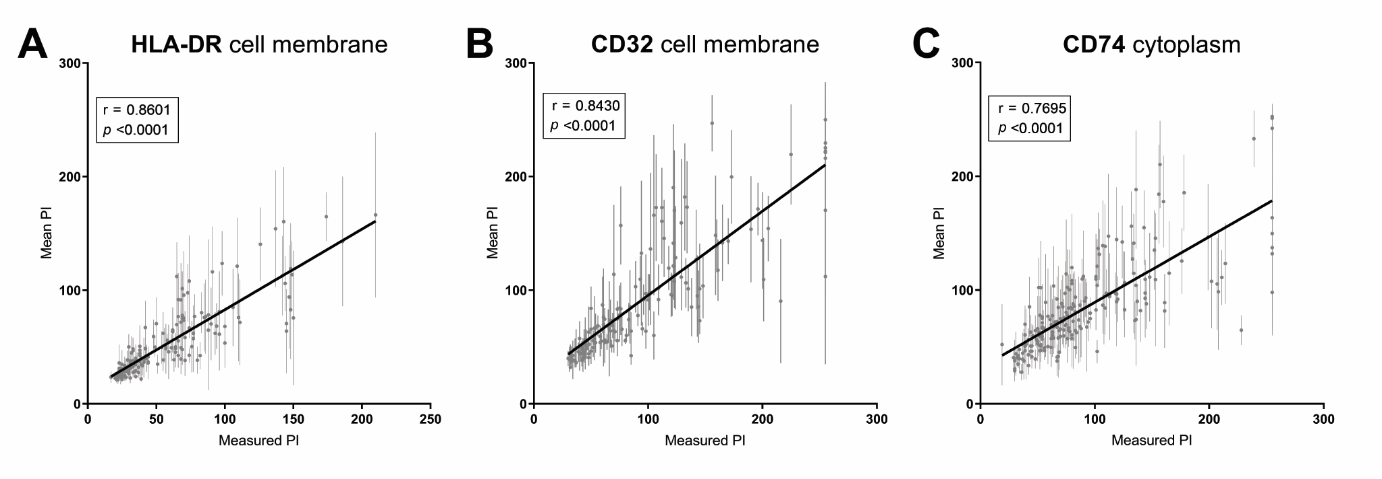
**

**Supplementary Figure 1**: Validation of single point intensities as measures of marker of interest abundance

10-point validation counting was carried out for cell membrane markers HLA-DR (A) and CD32 (B), and cytoplasmic marker CD74 (C). The original single measured point intensities were correlated with the mean point intensities from the 10-point validation using Pearson’s correlations. Point shown are mean point intensity ± SD against single measured point intensity (n = 148). Abbreviation: PI, point intensity.


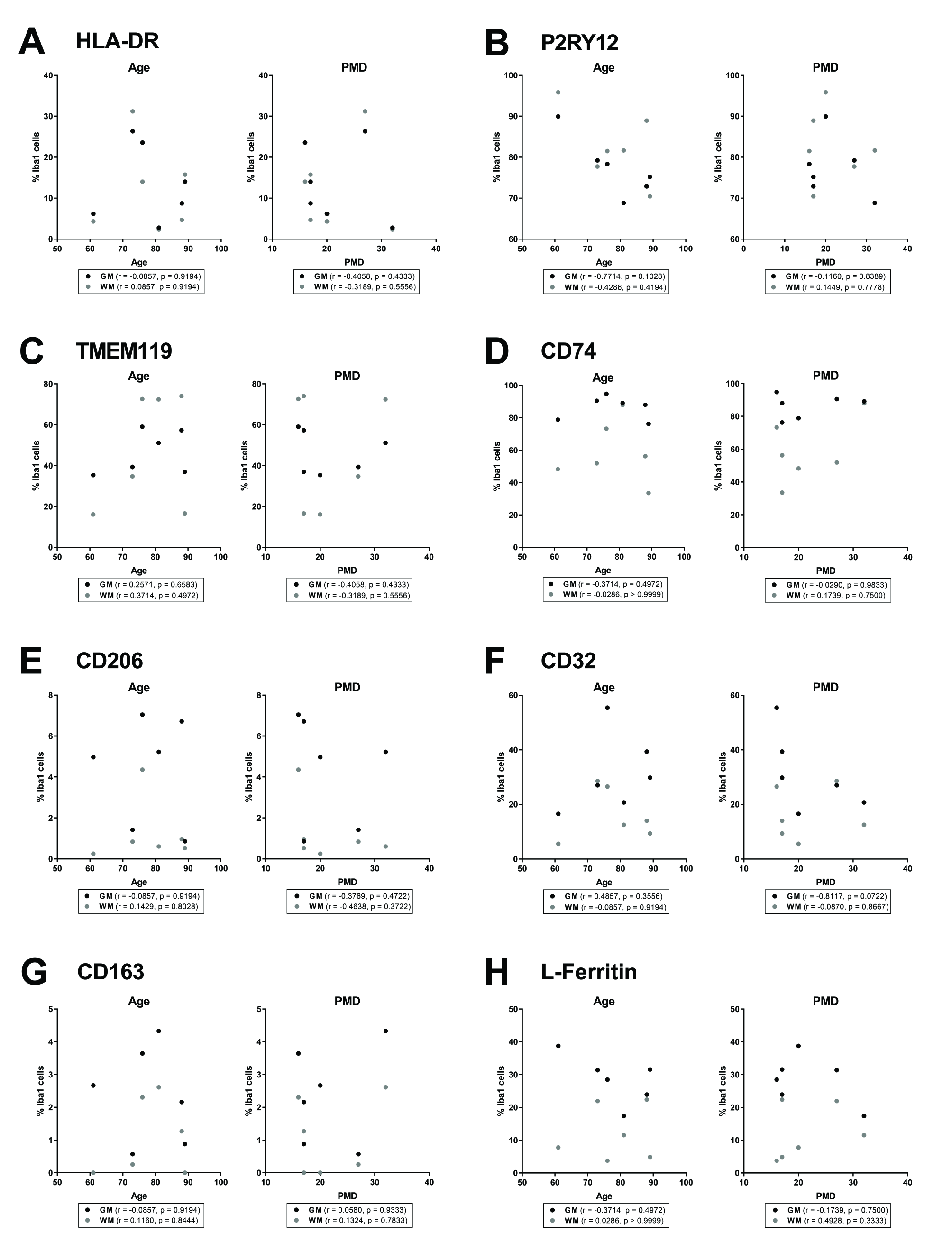


**Supplementary Figure 2**: Correlations of marker of interest abundances with age and post-mortem delay

Iba1-positive cells were manually counted in the human middle temporal gyrus and the proportion immunoreactive for MOIs was determined. The number of P2RY12 (A), TMEM119 (B), HLA-DR (C), CD74 (D), CD206 (E), CD32 (F), CD163 (G), and L-Ferritin (H)-positive cells (as a percentage of total Iba1-positive cells) in GM and WM were correlated with case age and post-mortem delay using Pearson’s correlations. Each data point shown represents a single case (total n = 6). Abbreviations: GM, grey matter; PMD, post-mortem delay; WM, white matter.

**
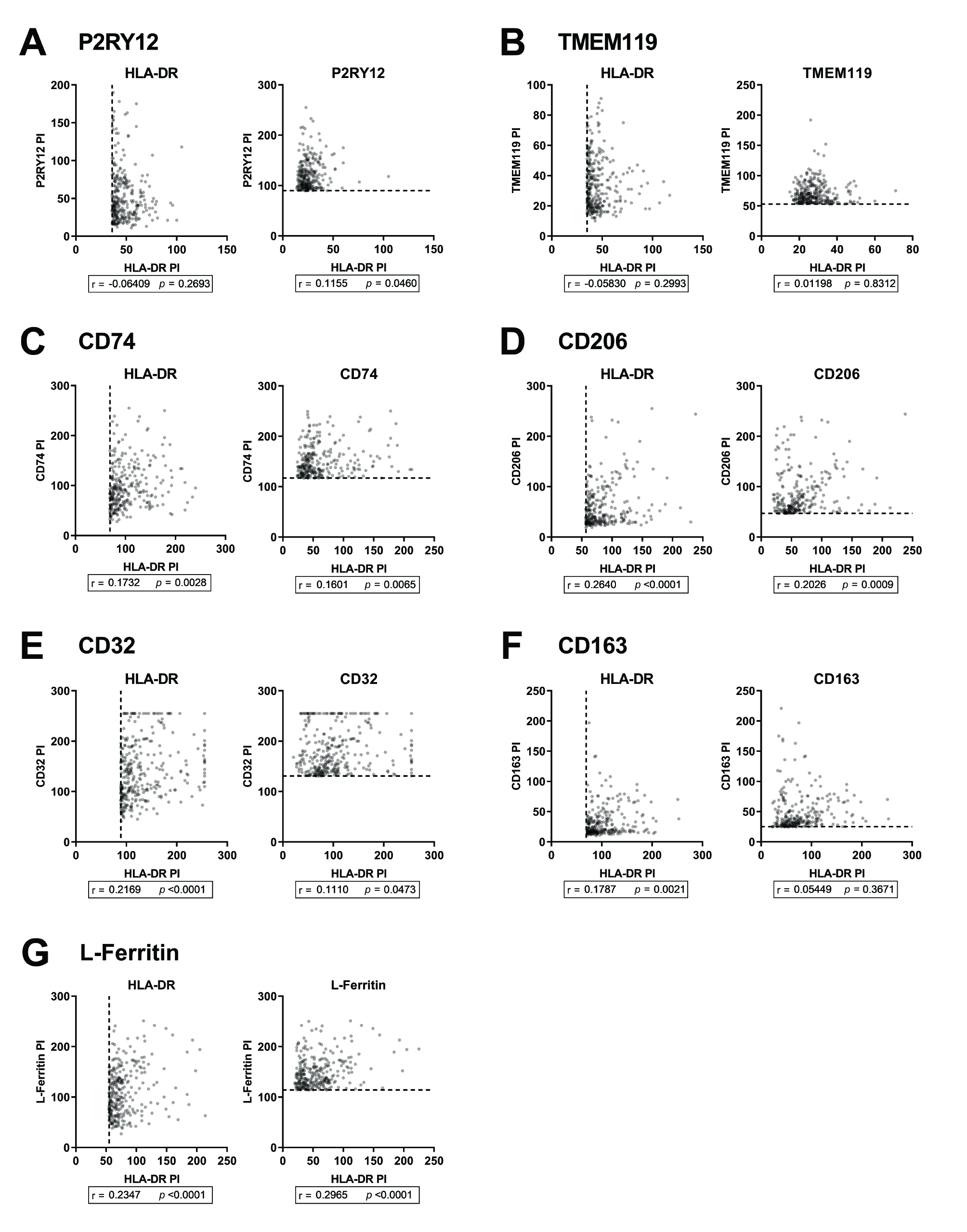
**

**Supplementary Figure 3**: Correlation analysis of top ten percent of marker of interest and HLA-DR point intensities.

One MOI was co-labelled with HLA-DR and pan myeloid cell marker, Iba1, in 10-µm thick normal human middle temporal gyrus sections. Iba1-positive cells in the grey matter were manually counted and the point intensities of the MOI and HLA-DR were measured. All Iba1-positive cell HLA-DR and MOI point intensities were pooled from all cases (n = 6). Pearson’s correlations were carried out between the point intensities of P2RY12 (A), TMEM119 (B), CD74 (C), CD206 (D), CD32 (E), CD163 (F), or L-Ferritin (G) and HLA-DR with only the top ten percent of HLA-DR or MOI point intensities. Abbreviation: PI; point intensity.
